# Remodeling the Bone Marrow Microenvironment – A Proposal for Targeting Pro-inflammatory Contributors in MPN

**DOI:** 10.3389/fimmu.2020.02093

**Published:** 2020-08-31

**Authors:** Jonas Samuel Jutzi, Ann Mullally

**Affiliations:** ^1^Division of Hematology, Department of Medicine, Brigham and Women’s Hospital, Harvard Medical School, Boston, MA, United States; ^2^Department of Medical Oncology, Dana-Farber Cancer Institute, Harvard Medical School, Boston, MA, United States; ^3^Cancer Program, Broad Institute, Cambridge, MA, United States

**Keywords:** MPN, JAK2, CALR, MPL, inflammation, megakaryocytes, monocytes, mesenchymal stromal cells

## Abstract

Philadelphia-negative myeloproliferative neoplasms (MPN) are malignant bone marrow (BM) disorders, typically arising from a single somatically mutated hematopoietic stem cell. The most commonly mutated genes, *JAK2*, *CALR*, and *MPL* lead to constitutively active JAK-STAT signaling. Common clinical features include myeloproliferation, splenomegaly and constitutional symptoms. This review covers the contributions of cellular components of MPN pathology (e.g., monocytes, megakaryocytes, and mesenchymal stromal cells) as well as cytokines and soluble mediators to the development of myelofibrosis (MF) and highlights recent therapeutic advances. These findings outline the importance of malignant and non-malignant BM constituents to the pathogenesis and treatment of MF.

## Introduction

Myeloproliferative neoplasms (MPN) are a group of clonal malignant bone marrow (BM) diseases, originating from a hematopoietic stem cell (HSC) which acquired a MPN phenotypic driver mutation (i.e., in *JAK2*, *CALR*, or *MPL*), leading to constitutively active JAK-STAT signaling (1, 2). Although the pathogenesis of MPN is cell-intrinsic to hematopoietic cells, MPN cells also exert cell-extrinsic effects resulting in chronic inflammation that perturbs the BM niche, and which in turn contributes to the MPN phenotype and renders the niche less supportive of normal hematopoiesis (i.e., the malignant self-perpetuating niche) (3).

The three main MPN clinical entities are polycythemia vera (PV), displaying an increase in red blood cells, essential thrombocythemia (ET), presenting with increased platelets and primary myelofibrosis (PMF), showing fibrosis of the BM. Common features of MPN, most pronounced in myelofibrosis (MF) patients, are increased levels of pro-inflammatory cytokines, leading to chronically increased inflammation in the BM and resulting in constitutional symptoms (e.g., fatigue, weight loss).

Eradicating malignant MPN cells in patients has so far failed in settings other than allogeneic HSC transplantation and in a minority of patients with PV and ET treated with interferon (4, 5). Another, complimentary approach to break the vicious cycle of aberrant “cross talk” between malignant hematopoiesis and the BM microenvironment is to inhibit the secretion of pro-inflammatory cytokines in both malignant and non-malignant cell populations. This has the potential to limit the expansion of the malignant hematopoietic clone and slow down or even prevent MPN disease progression.

In this review, we focus on secreted pro-inflammatory factors of MPN, cell-autonomous and cell non-autonomous contributors to MPN as well as novel approaches targeting these factors.

## Cytokines and Soluble Mediators

A wide variety of immune-modulatory cytokines are elevated in MPN patients, including IL-1, IL-6, IL-8, IL-10, IL-11, IL-17, TNFα, and TGFβ (6–10). Most of the listed cytokines are either pro-inflammatory like IL1 or directly pro-fibrotic factors as in the case of transforming growth factor beta (TGFB), with the exception of IL-10 which has an anti-inflammatory role. While MPN is caused by genetic mutations in HSC, its progression is often driven, at least in part, by inflammation. Cytokines like IL-1, IL-6, and TGFβ have been identified to contribute to the pathogenesis of fibrosis and osteosclerosis of the BM (11). NFκB signaling is frequently increased in MPN patients and required for downstream expression of pro-inflammatory cytokines like IL-8 (12). IL-8 itself has been implicated in leukemic transformation in MF patients (10, 13). In patients with PV, IL-12 levels correlate with hematocrit levels, IL-1β correlates with leukocytosis, and IFNα as well as IFNγ with the risk of thrombosis. Lastly, MIP1β has been shown to be associated with shorter overall survival (14). In patients with ET a recent longitudinal study on more than 400 patients described an ET-specific inflammatory cytokine signature compromising CCL11 (eotaxin), CXCL1 (GROa), and epidermal growth factor (EGF) (15). Finally, chemical mediators such as reactive oxygen species (ROS) have also been associated with inflammation-induced genomic instability and DNA damage in JAK2^V617F^-positive MPN patients, and this topic has been reviewed elsewhere (16–18). In summary, it is now apparent that circulating cytokines are perturbed in MPN, not just in established MF, but also in PV and ET. Furthermore, these studies provide indirect evidence that inflammation is not just an “innocent bystander” in MPN, but also contributes to clinically relevant outcomes.

## Cellular Contributors to Inflammation

Inflammation is increasingly thought to play an important role in the development of chronic myeloid malignancies like MPN as well in progression to acute leukemia (19–22). Several different cell types are involved in initiating and/or perpetuating inflammation. In this review, we address four major cellular contributors of inflammation in the context of MPN.

### Hematopoietic Stem and Progenitor Cells

Recent advances in single-cell approaches have uncovered MPN-specific lineage-trajectories and transcriptional programs. In a recent study Psaila et al. combined single-cell RNA sequencing (scRNA-seq) with targeted single-cell mutational analysis on the same MPN cell (TARGET-seq) in MF (23). They found that *JAK2*-mutant MF HSPCs are biased toward the megakaryocyte-lineage from an early HSC stage, where megakaryocytic surface markers (e.g., CD41) are absent (23). In an earlier paper, Nam et al. also linked genotyping of expressed genes to their transcriptional profile [Genotyping of Transcriptomes (GoT)] (24). They performed GoT on CD34^+^ cells from patients with *CALR*-mutated MPN and found upregulation of NFKBIA and CXCL2 specifically in *CALR*-mutated HSPCs (24). Together, these studies indicate that MPN-specific pro-inflammatory transcriptional programs are activated early in the hematopoietic hierarchy in both *JAK2*-mutant and *CALR*-mutant MPN.

### Monocytes

Studying leukocytes gained attention in MPN as it became apparent, they are not a mere by-product of the malignancy but also impact clinical outcomes. Leukocytosis is an independent risk factor for thrombosis (25) and there is growing evidence that activated monocytes contribute to MPN morbidity through secretion of pro-fibrotic cytokines and pro-thrombotic factors (26), regardless of their own mutational status (27). It has been shown that MPN patients with thrombotic events had higher levels of CD25^+^ monocytes compared to patients without thrombosis (26). MPN monocytes show an over-reactivity in their production of TNFA as a consequence of an impaired response to anti-inflammatory IL10, frequently elevated in MPN patients (27). The underlying mechanism is still unknown, however, this failure in response was seen in JAK2^V617F^-positive and -negative monocytes from the same patients (27). A recent study by Fisher et al. found that classical CD14^+^CD16^–^ monocytes, but also CD14^+^CD16^+^ inflammatory monocytes as well as CD14^–^CD16^+^ non-classical monocytes, all contribute to the overproduction of cytokines in MF, including TNF, TGFβ, and IL-10 amongst others (28). In the ET-specific inflammatory cytokine signature described by Øbro et al., they identified monocytes as the predominant producer of CXCL1 (GROa) in patient samples (15). Together, these findings highlight the non-cell autonomous contributions of monocytes in MPN.

Fibrocytes are a distinct cell population (related to monocytes), arising in the BM and displaying characteristics of both mesenchymal and myeloid hematopoietic cell origin. Human fibrocytes express stem cell markers (CD34) and monocyte markers (CD14, CD11) as well as markers of stromal cells (collagen I, III) (29, 30) and secrete the extracellular matrix (ECM) proteins, collagen I and vimentin (31, 32). A study by Verstovsek et al. found that MF patients carry clonal fibrocytes producing collagen and fibronectin, which are key constituents of fibrous tissue in the BM of MF patients (30). Transplanted MF BM displayed a fatal MF-like phenotype in immunocompromised mice. Interestingly, treatment with serum amyloid P (=pentraxin 2), a known fibrocyte inhibitor, reduced BM fibrosis and prolonged survival (30). Another recent study found that BM-derived fibrocyte-precursor CD14^+^/CD34^+^ monocytes, obtained from MF patients were able to induce an MF-like phenotype in immunocompromised mice (33). Mice developed splenomegaly, reticulin fibrosis and megakaryocyte clustering (33). Moreover, under TGFB stimulation, fibrocytes lose their CD34^+^ and CD45-positivity and express smooth-muscle actin (a-SMA) (34), making them myofibroblast-like. Myofibroblasts are contractile, fibrosis-causing and collagen-secreting cells (35).

Taken together, these studies support the idea that monocytes and their derivates contribute to MF and are therefore potential candidates for future targeted therapies.

### Megakaryocytes

Megakaryocytes are increased in the BM of MF patients, resulting in the overproduction of pro-fibrotic cytokines and are therefore considered to be a major cellular driver of BM fibrosis (36–39). Woods and colleagues found activation of Jak/Stat signaling and expansion of megakaryocytes in Jak2^V617F^-Pf4iCre mice, which was developed to restrict Cre recombinase-mediated excision to megakaryocytes and its progeny (40). Using Jak2^V617F^-Pf4iCre mice, Zahn et al. showed that Jak2^V617F^-mutant megakaryocytes promote the expansion of hematopoietic stem and progenitor cells (HSPCs) in mice (41). A recent study verified that the expansion of HSPCs was due to constitutively active thrombopoietin/MPL signaling, resulting in increased megakaryocytes, and causing HSPC expansion through cell non-autonomous mechanisms (42). Moreover, expression of mutant Jak2 in megakaryocytes was sufficient to induce fibrosis and erythropoiesis, the latter due to increased levels of IL6 (42). This finding supports other studies showing a cell non-autonomous effect of the *Jak2*-mutant clone on wildtype cells (40). While there have been earlier reports suggesting that Pf4iCre does not restrict recombination solely to megakaryocytic-lineage cells (i.e., “leaky” recombination in other lineages) (43, 44), a recent study by Mansier et al. investigated this specifically in the context of Jak2^V617F^. Using Pf4iCre, the authors detected Jak2^V617F^ expression in a fraction of HSCs (45), suggesting that recombination in HSC cannot be excluded as a contributing factor to some of the findings in the earlier studies focused on the cell non-autonomous effects of megakaryocytes in Jak2^V617F^-driven MPN (40–42).

Comprehensive single-cell sequencing is revolutionizing the field of hematology by providing high-resolution profiling of hematopoietic cell populations and by re-defining the hematopoietic hierarchy in normal and malignant hematopoiesis (46–48). Gene set enrichment analysis of megakaryocyte precursors (MkPs) revealed enrichment of inflammatory pathways in MF MkPs as compared to MkPs from healthy donors (HD) (23). A subset of these MkPs (displaying similar expression profiles between HD and MF MkPs), showed high expression of known mediators of MF (PDGA, CCL5, and CXCL5) (23). Most MF MkPs however, had a distinct transcriptional profile from HD MkPs, indicating the expansion an aberrant megakaryocyte population in MF (23). Some MkP populations display selective expression of AURKA, a kinase that has previously been proposed as a therapeutic target in MF (39). In addition, there have been several studies focused on the contributions of platelets to inflammation in MPN, a topic that was recently reviewed by Oyarzún and Heller (49).

In summary, megakaryocytes have been shown to contribute to MPN pathology, by fueling the proliferation of malignant and wildtype cells through cell non-autonomous effects, while also promoting inflammation and MF.

### Mesenchymal Stromal Cells

It has been appreciated that BM mesenchymal stromal cells (MSCs) contribute to inflammation (3) and to the pathogenesis of MF (50–52). Importantly, it has been shown that MSC do not harbor JAK2^V617F^ (30, 53–55).

In experimental mouse models, perturbation of MSCs has been shown to induce BM fibrosis by indirectly influencing HSCs, as in the case of deletion of the retinoblastoma gene (Rb), a cell-cycle regulator in hematopoiesis. A study by Walkley et al. showed that genetic knockout of Rb in the entire hematopoietic system using the inducible MxCre system leads to a myeloproliferative phenotype and extramedullary hematopoiesis (56). However, this was not the result of an HSC cell-intrinsic phenotype but due to cell-extrinsic Rb-dependent crosstalk between HSCs and the BM niche (56). Another example where perturbation of MSC in experimental mouse models induced MF is in mice deficient in the expression of the retinoic acid receptor gamma (RARγ^–/–^), specifically in the BM niche. Wildtype BM transplanted into RARγ^–/–^ mice showed an MPN phenotype mirroring several features of human MF (57), again highlighting the role of MSCs in driving MF phenotypes *in vivo*.

Specific subgroups of MSCs have been identified to be cellular drivers of BM fibrosis, including the Leptin receptor (Lepr) and Gli1^+^ MSCs (58, 59). Lepr^+^ MSCs differentiate into myofibroblasts in the context of thrombopoietin (TPO) overexpression-induced MF, accompanied by upregulation and secretion of proteins linked to MF (e.g., collagen) (58). Gli1^+^ and Lepr^+^ MSCs do not express the common hematopoietic surface marker CD45, highlighting a different process of myofibroblast differentiation as compared to monocyte-derived fibrocytes which are CD45^+^ (59). Blockade of the platelet-derived growth factor receptor a (Pdgfra), a driver of BM fibrosis in Lepr^+^ MSCs cells, strongly suppressed MSC growth. Conversely, Pdgfra overexpression increased MSCs and extramedullary hematopoiesis. These findings highlight PDGFRA signaling as a potential therapeutic target in MF patients (58). Martinaud and colleagues performed whole transcriptome profiling of MSCs from patients with MF and from HD and found a clear pro-fibrotic and inflammatory signature in MSCs from patients with MF (60). MSCs from patients with MF overexpressed pro-inflammatory factors (e.g., TGFβ1, BMP2) and ECM components (e.g., glycosaminoglycans, chondroitin sulfate, and heparan sulfate) (50).

In summary, as the field has developed a better understanding of the cellular components of the BM microenvironment, this has led to a shift away from focusing solely on cell-intrinsic contributions to myeloid malignancies to a more holistic view of HSPCs in their BM niche.

## Therapeutic Targeting of Soluble Mediators, the Malignant Bone Marrow and Cellular Contributors of MPN-Driven Inflammation

Simplified, there are two main approaches to treating MF. Firstly, the eradication of the malignant hematopoietic clone and secondly, the modulation of cellular components and soluble mediators including through inhibiting signaling pathways in MF.

### Targeting Soluble Inflammatory Mediators

Inflammation plays a role in all MPN subgroups, most pronounced in MF patients. It has been shown that inhibiting specific cytokines like IL-1β or the NfkB pathway can either decrease hematopoietic cell growth *ex vivo* (61) or even diminish fibrosis *in vivo* (62). Targeting soluble mediators in MF patients serves predominantly to ameliorate constitutional symptoms and reduce frequent comorbidities like MF -associated anemia. In patients with MF, reduction of pro-inflammatory cytokines induced by treatment with the JAK1/2 inhibitor, ruxolitinib correlated with symptomatic improvement (63). More recently, Fisher et al., using mass cytometry, found a limited effect on the levels of pro-inflammatory cytokines in MF patients treated with ruxolitinib (28) with plasma cytokine levels remaining markedly abnormal despite JAK2 inhibition (28). Some of the elevated cytokines were responsive to *ex vivo* pharmacological inhibition of the NfkB and/or the MAP kinase signaling pathway (28), highlighting the importance of these pathways for future cytokine-directed therapies in MF.

Momelotinib, a JAK1/2 inhibitor, which also inhibits the activin A receptor type 1 (ACVR1) has shown significant improvement in anemia in treated MF patients (64, 65). It is thought that the anemia response may be mediated via an indirect mechanism resulting in suppression of hepcidin and releasing storage iron to promote erythropoiesis (66, 67). Another agent, currently in a phase II study for MF patients (NCT03194542), is luspatercept, a TGFB super family ligand-binding fusion protein which reduces downstream SMAD signaling, and acts as an erythroid maturation agent (68, 69). Notably, luspatercept recently gained FDA-approval for the treatment of anemia associated with beta-thalassemia and for myelodysplastic syndrome (MDS)-related anemia (NCT02631070, NCT03682536) (70, 71). INCB039110, a JAK1 inhibitor was tested in a phase II clinical trial for MF patients and aimed to reduce elevated cytokine levels to improve constitutional symptoms (72). Plasma pro-inflammatory cytokine levels (e.g., CRP, IL-6, VEGF) were significantly decreased in most patients. JAK2^V617F^ allele burden, however, was non-significantly changed (72). In about half of the patients, red blood cell transfusions could be reduced by 50% or more during the duration of the study, spleen volume was slightly decreased and effects on myelopoiesis were mild (72).

### Targeting Malignant Hematopoietic Cells

The first targeted therapy for MPN patients was introduced in 2011 when the JAK1/2 inhibitor ruxolitinib (INCB-018424) gained FDA-approval for the treatment of patients with intermediate and high-risk MF (13, 73). This approach led to a decrease in spleen size and reduction in constitutional symptoms and a better 5-year overall survival, however, ruxolitinib does not substantially reduce the JAK2^V617F^ variant allele fraction (74–77). Limitations in targeting JAK2 are caused by the dependency of normal hematopoiesis on JAK2, resulting in on-target toxicity in the form of anemia and thrombocytopenia in patients with MF treated with JAK2 inhibitors (77, 78). Fedratinib is a selective JAK2-kinase inhibitor which also showed significant reduction in spleen size and improvement in constitutional symptoms in patients with MF and was recently was FDA-approved as both a first line and second-line therapy (following ruxolitinib failure) in MF (79–82). Several other JAK inhibitors are currently in late phase clinical trials (e.g., momelotinib and pacritinib) and will likely gain FDA-approval also.

Targeting megakaryocytes selectively has shown efficacy in several preclinical and early phase clinical studies (38, 39). Three approaches regulating megakaryocyte maturation have shown benefits. First, anagrelide, a megakaryocyte maturation inhibitor (83), was shown to be effective in ET patients (84). Second, targeting AURKA which was recently shown to be differentially expressed in *JAK2*-mutant MkPs in MF (23), with alisertib (MLN8237) promoted megakaryocyte polyploidization and to reduced MF in preclinical studies (39) and has shown some benefits in MF patients (85). Third, bomedemstat (IMG-7289), an inhibitor of LSD1, an enzyme essential for platelet formation (86), was recently granted FDA fast-track designation for the treatment of ET patients (NCT04254978). In murine models of MPN, IMG-7289 has shown efficacy in reducing inflammation, splenomegaly and fibrosis, in addition to prolonged survival (87). IMG-7289 killed Jak2^V617F^-mutant cells selectively and synergized with Jak inhibition in pre-clinical MPN mouse models (87). Bomedemstat is currently in phase IIb clinical trials for MF patients (NCT03136185).

Recently, Psaila et al. showed differential increased expression of G6B in *JAK2*-mutant HSPCs in MF (as compared to wildtype HSPCs from the same patient) (23). G6B is an immunoreceptor tyrosine-based inhibition motif (ITIM)-containing inhibitory receptor, normally expressed exclusively on mature megakaryocytes in normal hematopoiesis (23, 88, 89). The authors identified *JAK2*-mutant HSPCs using G6B expression and validated this cell surface marker as a candidate for specifically targeting *JAK2*-mutant HSPCs in MF, using a bi-specific antibody (against CD34 and G6B), as a potential future novel therapeutic strategy (23).

As PMF is characterized by the progressive deposition of ECM proteins (90), another therapeutic approach is to normalize the composition of the ECM. Lysyl oxidases (LOXs) have been demonstrated to be important in this process by cross-liking collagens and elastins through deamination of lysins and hydroxylysins, resulting in a stiffer ECM consistency (91). Lysyl oxidases are expressed in immature megakaryocytes and downregulated in mature megakaryocytes but upregulated in MF patient megakaryocytes and in murine models of MF (38, 92, 93). Lysyl oxidase inhibition has shown efficacy in Gata1^low^ (38) and JAK2^V617F^ mouse models of MF (94–96). However a recent phase 2 study of simtuzumab, a monoclonal inhibitor of LOX2 did not reduce bone fibrosis in patients with MF (97).

In conclusion, more effectively targeting cellular components of malignant hematopoiesis in MPN remains an ongoing goal within the field.

### Targeting the Bone Marrow Niche

Therapeutically targeting the BM stroma has gained more attention in the treatment of MF (59, 98). As highlighted before, Gli1^+^ MSC were shown to be an important driver of MF in mouse models highlighting them as a potential therapeutic target. Gli1 as well as Ptch1 are known hedgehog (Hh) target genes, previously shown to be increased in MPN patients (99). Treatment with the Gli inhibitor, GANT61 in a JAK2^V617F^ MF mouse model reduced the expression of mediators of inflammation and fibrosis significantly (e.g., MMP9, CXCR4, endothelin 1) (59). Moreover, treatment also reduced Stat5 expression in JAK2^V617F^-mutant cells, thereby decreasing pro-inflammatory signaling in the BM and interrupting the self-reinforcing cycle of inflammation, myofibroblast differentiation and ECM deposition. *Ex vivo* treatment of primary human MPN MSCs with GANT61 reduced the expression of both a-SMA and GLI1 and increased apoptosis (as compared to vehicle treatment) (59). These findings suggest selective targeting of GLI1-positive myofibroblasts by the inhibitor, making it an attractive candidate for potential clinical use in MPN patients (59).

The NfκB pathway has been shown to be activated in JAK2 mutated MPN. Recently, a potential combinatorial therapeutic approach for MPN patients has been proposed, by targeting inflammation through reduction of NfκB activity using BET inhibition in combination with JAK inhibition (62). Using MPN mouse models, Kleppe et al. showed that increased NfκB activity in MPN is partly cell-extrinsic, highlighting the importance of targeting the BM microenvironment. The BET inhibitor, JQ1 showed potent anti-fibrotic effects and cooperated with Jak inhibition to ameliorate inflammation (62). Moreover, the NFKB pathway has also been shown to be upregulated in *CALR* mutated MPN HSPCs (24), suggesting that BET inhibition might also be effective in *CALR*-mutant MPN patients. Preliminary data using the BET inhibitor, CPI-0610 in MF patients either alone or in combination with ruxolitinib (MANIFEST study), showed beneficial effects. CPI-0610 alone or as ab add-on to ruxolitinib was well-tolerated and showed a reduction in BM fibrosis, spleen size and amelioration of anemia in MF patients (100, 101).

Taken together, these studies underscore the importance of treatment strategies for MPN that target the BM niche and highlight the potential for combinatorial targeting of both the malignant hematopoietic clone and the BM microenvironment to have enhanced efficacy.

## Conclusion

MPN comprise a group of clonal malignant hematopoietic disorders with common features such as myeloproliferation and systemic inflammation. While genetic driver mutation-specific targeted therapy is at the center of MPN research, recent evidence highlights the importance of regulating inflammation in MPN. Malignant and non-malignant cellular contributors such as megakaryocytes and monocytes, as well as the BM niche, promote disease progression and cause considerable morbidity. This emphasizes the importance of a broader approach to simultaneously inhibit several pathogenic contributors in MPN, with the goal of improving treatment outcomes. Ongoing studies will shed light on the efficacy (and potential toxicity) of combining targeted therapies with anti-inflammatory approaches for the treatment of MPN.

## Author Contributions

JJ drafted the manuscript. Both authors designed the outline for the manuscript and edited and approved the manuscript.

## Conflict of Interest

AM has received honoraria from Blueprint Medicines, Roche, and Incyte and receives research support from Janssen. The remaining author declares that the research was conducted in the absence of any commercial or financial relationships that could be construed as a potential conflict of interest.
